# Mosquitoes Reset Malaria Parasites

**DOI:** 10.1371/journal.ppat.1004987

**Published:** 2015-07-02

**Authors:** Philip J. Spence, Thibaut Brugat, Jean Langhorne

**Affiliations:** 1 Institute of Immunology and Infection Research, and Centre for Immunity, Infection and Evolution, University of Edinburgh, Edinburgh, United Kingdom; 2 Francis Crick Institute, Mill Hill Laboratory, London, United Kingdom; The Fox Chase Cancer Center, UNITED STATES

Serial blood passage of *Plasmodium* universally increases parasite virulence, which can be reversed by mosquito transmission. How mosquitoes reset *Plasmodium* virulence has been unknown. We have shown that mosquito transmission modifies expression of *Plasmodium* subtelomeric multigene families, including those that code for variant surface antigens (VSA), and transforms the systemic immune response to blood-stage infection. In this way, the mosquito regulates malaria disease severity. Here, we present a model in which expression of multigene families is reset by epigenetic reprogramming of *Plasmodium* within the mosquito. This prepares the malaria parasite for entry into a new unknown host and transforms the early parasite–host interactions that shape disease severity. Studying the molecular mechanisms that operate outside the human host to regulate *Plasmodium* virulence is therefore a priority.

## Historical Perspective

It has been recognised for decades that serial blood passage of *Plasmodium* through rodents, primates, or humans universally increases parasite virulence. In 1917, induced malaria was first used as pyretic therapy for neurosyphilis, with *Plasmodium vivax* routinely inoculated to elicit a mild form of disease. Yet passage through the human host elevated parasitaemia and exacerbated disease, increasing the requirement for chemotherapeutic intervention [1]. Blood passage of *Plasmodium knowlesi* or *Plasmodium cynomolgi*, whether through human volunteers or nonhuman primates, similarly elevated parasite densities and disease severity [2–4]. And serial blood passage of every rodent malaria parasite species increased parasitaemia and pathogenicity [5–8]. On the other hand, it has been assumed for decades that mosquito transmission resets *Plasmodium* virulence [8]. At the Horton Mental Hospital, a pioneering centre for malaria therapy, *Plasmodium* strains were maintained by mosquito transmission to preserve their clinical and parasitological features [9]. Nevertheless, direct evidence that mosquito transmission resets *Plasmodium* virulence, and a mechanism to explain this phenomenon, have been missing [6,10,11].

## Mosquito Transmission Resets *Plasmodium* Virulence

We have recently shown that mosquito transmission modifies gene expression in blood-stage malaria parasites and in this way resets *Plasmodium* virulence [12]. Whereas serial blood passage of *Plasmodium chabaudi* leads to hyperparasitaemia and severe disease in laboratory mice, mosquito transmission of serially blood-passaged parasites leads to a low-grade, chronic, recrudescing infection with minimal pathology. Attenuation of virulence is not parasite clone- or dose-dependent and therefore cannot be explained by bottlenecking during mosquito transmission [13]. Instead, attenuation of the blood-stage parasite is dependent upon host genotype [14] and an intact host immune response [12] and associates with increased expression of *Plasmodium* subtelomeric multigene families, including those that code for VSA (Box 1). Mosquito transmission therefore modifies expression of parasite virulence genes and transforms host immunity in the pathogenic blood-stage of infection. As such, the mosquito vector both transmits malaria and regulates disease severity.

**Box 1. Mosquito Transmission Modifies Expression of *Plasmodium* Virulence Genes in Human Malaria**

Transcriptional profiling [21] and proteomic analysis [22] of cultured *Plasmodium falciparum* demonstrates that the diversity and magnitude of *rifin* and *var* gene expression is increased in sporozoites (isolated from mosquito salivary glands) as compared to merozoites, trophozoites, or gametocytes. Furthermore, 53 of 59 *var* genes were transcribed in a single human volunteer infected with *P*. *falciparum* by mosquito bite just five days after merozoite egress from the liver [23], and diversity of *var* gene expression has been shown to decrease in human volunteers after blood passage [24]. Collectively, these data support a model in which expression of *Plasmodium* subtelomeric multigene families is increased as parasites transit through the mosquito and subsequently decreases with time elapsed from the vector.

## Epigenetic Reprogramming of *Plasmodium*

**Fig 1:**
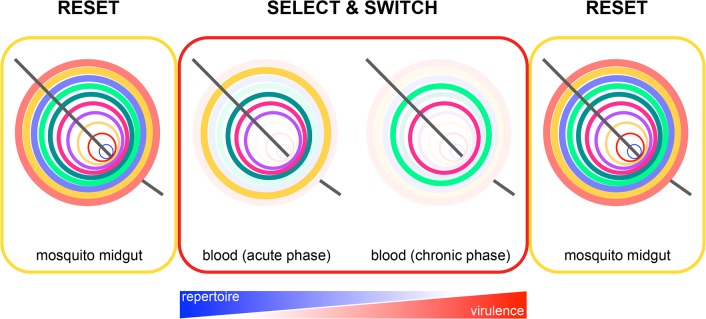
Epigenetic reprogramming of *Plasmodium* within the mosquito. Reset: expression of subtelomeric multigene families is reset in the mosquito by epigenetic reprogramming of the zygote. This ensures that a parasite population will always express all multigene family members from the start of the erythrocytic cycle and gives merozoites the best possible chance of establishing a blood-stage infection every time they emerge from the liver (e.g., it may be beneficial to express all VSA upon liver egress, as a malaria-experienced host will have pre-existing antibodies that recognise a broad repertoire of variant antigens [23]). Select and switch: parasites that express (or switch to) multigene family members offering a survival advantage in their new host are retained, whereas parasites that silence these genes are lost through rounds of selection. In time, this leads to a parasite population expressing a narrow repertoire of multigene family members that promotes survival and chronicity. The further into the chronic phase of infection, the better adapted are parasites to their host. *Plasmodium* virulence therefore increases, and the need to reset gene expression also increases. Reset: in preparation for entry into the next host, all chromatin marks are again erased following gamete fusion. This model of gene expression provides a general mechanism by which all *Plasmodium* subtelomeric multigene families could be regulated by the mosquito.

By recognising this key function of the mosquito, new research avenues open that can accelerate our understanding of the pathogenesis of human malaria. It is first important to delineate where, when, and how mosquito transmission modifies expression of *Plasmodium* virulence genes. This is likely to be a consequence, at least in part, of necessary changes in gene expression for progression through each step of the life cycle in both vector and host [15]. However, epigenetic reprogramming [16] of *Plasmodium* provides a mechanism by which expression of virulence genes could be reset within the vector. Heritable chromatin modifications control transcription of subtelomeric multigene families in the blood-stage parasite [15] and can thus promote adaptation of malaria parasites to their host [17]. Nevertheless, global erasure of epigenetic marks following gamete fusion in the mosquito could reset expression of multigene families and thus prepare *Plasmodium* for entry into a new unknown host (Fig 1).

Resetting *Plasmodium* gene expression could be particularly important when transmission is seasonal, and parasites undergo an extended period of host adaptation in a chronically infected individual before their return to the mosquito. In this context, it is important to know whether parasite virulence increases in the chronic phase of infection, as has been observed in human volunteers [3]. Thus, serial blood passage per se may not increase *Plasmodium* virulence; an alternative explanation is that virulence increases with time elapsed from the mosquito. This will be observed only in a new host and when mosquito transmission is bypassed.

## Immune Control of *Plasmodium* Virulence

By resetting *Plasmodium* gene expression, the mosquito can also control how blood-stage parasites elicit the systemic immune response in a new host. Mosquito transmission attenuates *P*. *chabaudi* virulence because merozoites that emerge from the liver induce an immune response that can rapidly control parasite growth without collateral damage [12]. This contrasts with the host response to serially blood-passaged parasites that causes severe immunopathology. Does increasing expression of *Plasmodium* VSA explain how the mosquito can transform the elicited host immune response? Or does mosquito transmission change the context in which blood-stage parasites initiate host immunity (e.g., by modifying invasion, cytoadherence, or sequestration)? Furthermore, it remains possible that immune priming and/or regulation during the pre-erythrocytic stages of infection can subsequently modify the systemic immune response to the blood-stage parasite. In all scenarios, the early immune response, elicited in the context of a mosquito bite, can shape malaria disease severity. In turn, the developing immune response is likely to influence expression of *Plasmodium* virulence genes and could therefore also directly regulate parasite pathogenicity.

## Improving Models of Malaria

Mosquitoes reset malaria parasites and can be used to strengthen the relevance of mouse models to human malaria. We should therefore aim to initiate experimental infections by the natural route of transmission wherever possible. We should also strive to study combinations of vector, parasite, and host that exist in nature to validate or improve our current experimental systems. Mouse models are important for interrogating the pathogenesis of malaria because they can answer research questions that cannot be addressed directly in humans. Moreover, relevant mouse models can act as a bridge between human studies. For example, vector regulation of *Plasmodium* virulence was first observed in human volunteers and subsequently reproduced and delineated in mice; the molecular mechanisms that operate within the mosquito to regulate *Plasmodium* virulence can now be dissected with human malaria parasites.

To this end, inoculation of human volunteers with *Plasmodium* is a powerful experimental model [18,19]. In this setting, it is possible to look for evidence of epigenetic reprogramming of *P*. *vivax* in laboratory-reared anopheline mosquitoes fed on infected volunteers. Interrogating expression and regulation of subtelomeric multigene families in gametocytes as they circulate, transmit, and then pass through each developmental checkpoint of sporogony is a priority. So, too, is examining how route of transmission influences the systemic host response to blood-stage infection. For this, the immune response to *P*. *falciparum* can be compared in peripheral blood obtained from human volunteers infected via mosquito bite versus direct inoculation of blood-stage parasites (isolated just 6–8 days after liver egress [20]). Nevertheless, mice are absolutely required to observe the interactions between parasites and the immune system that shape disease severity because these interactions occur in tissues, such as spleen. We should therefore aim to identify mouse models that share a common immune signature of infection in whole blood with human malaria and use these models to delineate the immune response to *Plasmodium* in relevant tissues.

## Concluding Remarks

A mosquito is not simply a flying syringe. Mosquitoes reset malaria parasites in preparation for entry into a new unknown host and thereby regulate *Plasmodium* virulence. Furthermore, they are a mixing pot for the generation of new recombinant parasites and can thus transmit previously unseen virulent strains. By studying events within the mosquito, we will accelerate our understanding of malaria disease severity.
